# The Symptomatology and Pathology of Exophthalmic Goitre1Read at the twenty-eighth annual meeting of the Medical Association of Central New York, at Syracuse, October 15, 1895.

**Published:** 1896-05

**Authors:** William C. Krauss

**Affiliations:** Buffalo, N. Y., Professor of nervous diseases, Medical Department of Niagara University; 382 Virginia Street


### THE SYMPTOMATOLOGY AND PATHOLOGY OF EXOPHTHALMIC GOITRE.[1]

### (BASEDOW'S DISEASE—GRAVES'S DISEASE.)

BY WILLIAM C. KRAUSS, M. D., Buffalo, N. Y.,
Professor of nervous diseases, Medical Department of Niagara University.

EXOPHTHALMIC goitre typically developed in an individual, is not at all difficult of diagnosis, although the treatment is somewhat discouraging and long-continued. It is, therefore, not my purpose to dwell upon typical cases of this affection today or to review with you the old-time and worn-out methods of treatment, but to call your attention to the "*formes frustes*," or illy-developed cases, and to report my manner of treating this disease. Before speaking of the *formes frustes* it might be well to review briefly the chief points of importance of this disease, especially the progress made in the past ten years, regarding its symptomatology, pathology and treatment. Exophthalmic goitre was first studied by Graves, the famous Dublin physician, in 1835; later by von Basedow, a celebrated physician of Merseburg, Germany, in 1840. It was found most commonly in women of middle age, although cases are on record where it was found in young girls, also showing some disposition to hereditary transmission, as evidenced by a case reported by Déjèrnie, where for four generations it remained a

1. Read at the twenty-eighth annual meeting of the Medical Association of Central New York, at Syracuse, October 15, 1895.

family disease. I have seen mother and daughter affected several times, but could trace it back no further. Oesterreicher reports where eight out of ten children in one family suffered, in various degrees and at various ages. It is met with less frequently in males, the proportion being five to one. Whether this great disproportion is due to the fact that the female is more often the victim of the emotions, sudden terrors or prolonged distress, would be difficult to say, but in my experience with this disease, those of the male sex afflicted were more or less effeminate in their ways and lacked those qualities which characterise the sterner sex.

SYMPTOMS OF EXOPHTHALMIC GOITRE.

| | | |
|---|---|---|
| *Primary or Cardinal Symptoms...* | | Tachycardia.<br>Goitre.<br>Exophthalmus.<br>Tremor. |
| *Secondary or Concomitant Symptoms.* | Of Digestive Tract.... | Vomiting.<br>Diarrhea.<br>Bulimia.<br>Icterus. |
| | Of Respiratory Tract.. | Cough.<br>Frequent respiration.<br>Dyspnea.<br>Angina. |
| | Of the Eye .......... | V. Græfe's sign.<br>Möbius' sign.<br>V. Stellwag's sign.<br>Becker's sign. |
| | Of the Nervous System | Nervousness.<br>Emotional states.<br>Neuralgias.<br>Epileptiform convulsions.<br>Psychoses. |
| | Of the Cutaneous System............... | Vitiligo, urticaria.<br>Pigmentation.<br>Hyperidrosis.<br>Flushings and sensation of heat.<br>Vigoroux's sign. |
| | Of the Urinary Tract.. | Polyuria.<br>Albuminuria.<br>Glycosuria. |
| | Of the Genital Organs. | Menstrual disturbances.<br>Impotence. |
| | Of Diverse Origin.... | Anemia.<br>Edema.<br>Cachexia.<br>Giving way of legs. |

These are the primary or cardinal and concomitant symptoms, and from their diversity and widespread dissemination show that the disease is not local or circumscribed, but one affecting several of the great systems of the body.

The cardinal symptoms, as tachycardia goitre and exophthalmus, were for years regarded as characteristic, and the triad always stood for the one disease. Pierre Marie, of Paris, was the first to point out that accompanying these symptoms there was most always present a tremor, resembling closely the tremor of mercurial poisoning, and in recent times this symptom has been looked upon as one of the chief or cardinal symptoms. Of the cardinal symptoms, the tachycardia is the most important, and for months, even years, may be the only evidence of a latent Basedow. The pulse may reach 200 per minute, but, as a rule, varies between 120 to 140 beats, is systolic, soft and small. The precordia, even the whole left side of the chest, will, in pronounced cases, reverberate in consonance with the heart-beats, giving rise to feelings of anxiety, suffocation and distress. As these symptoms continue, becoming more pronounced on exertion and excitement, that *bête noir* "nervousness" will, if not already present, make its presence known and the patient will consult her physician, and receive the information, in nine cases out of ten, that she is suffering from *nervousness*, if she be a working girl or woman; *nervous prostration*, if a society woman, and *neurasthenia*, if belonging to the XX. century or "New Woman" league. She will wander from physician to physician without obtaining relief, and then wonder whether or not doctors are what they are usually cracked up to be. The struma or goitre appears at a later stage than the tachycardia, grows slowly and uniformly, is soft, vascular and pulsating, and is present almost as frequently as the tachycardia. The stethescope reveals a systolic murmur, while palpation with the palm of the hand gives a thrill or purring. Of all the symptoms of this disease, the goitre will be most difficult to recognise in our women patients, unless you are looking especially for it, not because it is so illy defined or diffused, but because of its retreat behind sky-scraping linen collars, collar bands and other paraphernalia with which our lady friends so adroitfully and tastefully decorate their throats. It will not suffice to ask whether the throat is swollen or not; you must yourself with your fingers feel for enlargement of the thyroid, and, having found it, persevere in maintaining the fact, even in spite of the arguments of your patient to the contrary. The exophthalmus is less often present than the struma or tachycardia, is usually bilateral and easily overlooked, partly because of ignorance of the physician as to the patient's former appearance, and secondly, when not especially well marked, may resemble the "full, open eye" we so delight to see in a woman. Sometimes this symptom is so pronounced that the tendinous insertions of the eye muscles are visible, and in rare cases where the eye was forced out of the socket. The vision and retina remain undisturbed, and where the exophthalmus is not extreme, no pain is experienced by the patient.

The tremor which is now regarded as one of the cardinal symptoms is found present in almost every case, may affect the whole body, or the extremities, or the head and shoulders only. The tremor is of the rapid variety, ranging from eight to ten oscillations per second, increasing in intensity under excitement until it resembles the coarse movements so characteristic of chorea.

These four symptoms, when well developed, leave no doubt in the physician's mind regarding the diagnosis—they are pathognomonic. The secondary, or concomitant, symptoms are less characteristic, and, existing alone without the cardinal symptoms, cannot determine a case.

The digestive disturbances are perhaps the most annoying to the patient, and give the physician the most trouble and best opportunity for reviewing his works on therapeutics. The vomiting is at times uncontrollable, but must not be confounded with the projectile vomiting of brain lesions. It is not of the projectile variety, occurs after meals and attended with considerable nausea. The diarrheas are most distressing, very frequent and sudden, the stools are of watery consistency and very debilitating to the patient.

The respiratory symptoms consist of a hoarse, dry, convulsive cough ; dyspnea and frequent respiration, owing, perhaps, as Bryson has recently demonstrated, to a lack of chest expansion during respiration.

The secondary eye symptoms are perhaps the result of the exophthalmus ; certain it is that the greater the bulging the more pronounced will be the signs of v. Graefe, Möbius and Stellwag. v. Graefe's sign is the refusal or tardiness of the upper eyelids to follow the eyeball when slowly directed downward. Möbius's sign consists of an insufficiency of convergence, due to some disturbance of the internal recti muscles. If a patient be asked to look at a distant point, then at a near point, one eye will converge while the other diverges.

Stellwag's sign consists of an almost complete absence of the involuntary winking of the lid, and is quite conspicuous, especially since the voluntary movements can be made as well as before. The lids are retracted, the eyes consequently appearing abnormally large, even though there exists but slight protrusion of the bulbs. These disturbances, Graefe's and Stellwag's, are thought to be due to some affection of the small muscles of Müller, which are supplied by the sympathetic nerve. The sensibility of the cornea is diminished, is dry and lusterless, owing to the want of moisture on the ball, the normal quantity of the lachrymal secretion not being sufficient on account of the undue evaporation taking place, because the two lids are so far apart and winking only rarely occurs.

The ophthalmoscope reveals a normal fundus, with the exception of a spontaneous pulsation of the retinal vessels. This phenomenon was first discovered by Becker.

The nervous symptoms proper are the most developed of all and exist at the very earliest moment, as we shall see when we study the *formes frustes*. Nervousness is personified in every look, thought, movement and gesture of the patient. In no other affection of the nervous system is it of so virulent a type, so uncontrollable and so little amenable to treatment as a symptom. It shows itself in various forms, as irritability, distraction, forgetfulness, petulance, snappishness, and transforms an otherwise sweet-tempered person into an irascible, passionate, fretful, choleric and splenetic individual. It is often impossible to obtain information from them, on account of evasive replies, wrong interpretation of the questions and inability of the patient to remember his past feelings. Sometimes psychical disturbances supervene, as mania, melancholia and hallucinatory insanity, and in such cases the prognosis is most grave. The almost universal presence of the nervous condition should put a physician on his guard, and when, after a course of treatment of a "nervous body," no beneficial results are obtained, to conclude that the case is not simply nervousness, but that he has some deeper-seated affection on his hands.

The cutaneous symptoms include obstinate urticaria, vitiligo, dirty discolorations of the skin, pigmentation, covering the entire body or only localised. I saw a case in Westphal's clinic of this disease, where the skin was of the dirty bronze color, and in reality turned out to be Addison's disease, combined with exophthalmic goitre. The hair turns gray prematurely, falls out rapidly and is dry and brittle. The patient is greatly discomforted by profuse perspiration, either affecting the whole body, or the hands, head, or feet only. This hyperidrosis sometimes affects one side of the body only, and is at times most pronounced during sleep. This saturation of the skin is thought to be the cause of another quite characteristic symptom, discovered by Vigoroux at the Salpêtrière, in Paris — namely, that the electro-motive resistance of the skin was much less in patients affected with exophthalmic goitre than in healthy individuals. For instance, the electro-motive force of one Leclanché cell might register one milliampère on the galvanometer, while in a case of exophthalmic goitre it would register two to five milliampères.

Polyuria, albuminuria and glycosuria are frequently observed, attended with great thirst and feeling of intense heat. Disturbances of menstruation, as dysmenorrhea and amenorrhea are often noted in the female, while in the male, sexual impotence sometimes develops. Cachexia, often with rise of temperature, is present in many cases, giving rise to feelings of heat, and on the least exertion, mental or physical, the patient is bathed in perspiration. The cachexia, with increase of bodily temperature, resembles very much an ordinary febrile affection, but differs from a true fever in that the urine is free from those qualities and constituents which characterise the febrile state.

Local edemas affecting the lower extremities, eyelids or other unusual parts of the body, have been frequently reported, they being of nervous origin. In very severe cases there may develop anasarca, the result of heart weakness, leading to an unfavorable termination. Other symptoms have from time to time been noted by observers, but as they are met with so rarely I will not detain you with their enumeration.

Trousseau, the great French clinician, in studying many of these cases of exophthalmic goitre, found that a large percentage did not reach full development or, in other words, did not show the three cardinal symptoms unfolded to the same degree or intensity. He found that in some patients certain symptoms would be well marked, while others would be hardly perceptible or even absent, and yet he called such cases genuine.

This was contrary to the teaching in vogue up to that time, which insisted upon the undoubted presence of the goitre, tachycardia and exophthalmus. Trousseau accordingly proposed the term, *formes frustes*, meaning "abortive cases," for all such as were faulty in one or more of the cardinal symptoms, and as these cases are more thoroughly studied they are found to exist in relatively large numbers. Marie, under the guidance of the great Charcot, took up this subject a few years ago and found that many cases of so-called simple nervousness were, in fact, nothing less than the *formes frustes* of Trousseau. His studies awakened renewed interest, and as a result the *formes frustes* are more properly diagnosticated than ever before.

In these abortive forms there were generally recognised two prominent symptoms, to which was often added the third. The patients complained of nervous excitability and unstability, with a very rapidly pulsating heart or continuous palpitation. Insomnia and a nervous dyspepsia, along with the usual nervous accidents, misled physicians and do even to this day. Up to Marie's time the tremor had not been observed, but he recognised it in nearly all cases, and this has received universal recognition. This syndrome, nervousness, tachycardia and tremor, was found most often in the *formes frustes*, but other combinations are equally entitled to the same designation. In fact, any combination having one or more of the cardinal symptoms associated with one or more of the digestive, respiratory, ocular, secretory or genito-urinary symptoms, may very properly be designated *formes frustes*, and these forms the physician needs bear in mind. These cases are amenable to proper treatment as we shall see, and do not belong to that class of nervous affections, which physicians generally recommend any and all kinds of treatment, shifting from one drug or therapeutic measure to another until the patience and purse of the sufferer are taxed to the utmost.

*Pathology.*—This disease has, if observers can be believed, almost as many pathologies as symptoms, but I shall only discuss three of these theories today. Perhaps the most probable of these three possible theories of the pathogenesis of exophthalmic goitre is the one advanced by Köbens, and defended by Trousseau, Eulenburg, Oppenheim and many others, that it is due to some affection of the sympathetic nervous system. Certainly the majority of the symptoms are traceable to some lesion of this system, notably the tachycardia, perhaps the exophthalmus and goitre, the vasomotor, the secretory disturbances and other symptoms. Some are the result of irritation along the sympathetic, while others are due to paralysis of the sympathetic. My own view coincides with this theory, and until some more plausible theory is advocated I shall consider exophthalmic goitre as a disease of the sympathetic nervous system.

The second theory, supported by Sattler, Putnam, Mendel, Filehne and others, place the focal lesion in the medulla, more especially implicating the nucleus of the vagus nerve. Filehne, by transecting the corpora restiforme in animals, produced many of the symptoms of Graves' disease. Dardufi and Bienfait attained the same result. Hirt is inclined to accept this theory and places the chapter on this affection in the list of diseases of the vagus nerve. Another theory advocated by Möbius, and which has enlisted many supporters, especially during the past few years, is that it is the result of an abnormal activity of the thyroid gland, which, being absorbed by the system, produces toxic effects. This theory has had the effect of calling the surgeon to our help and removing the thyroid gland. Buschan collected 98 cases of thyroidectomy in this disease, with only 16 cures, while 14 per cent. of those operated upon died. Buschan, who has done excellent work in this field, believes it to be a functional disturbance of the whole nervous system, especially of the cerebro-spinal centers.

*Treatment.*—The treatment is as varied as are the theories regarding its pathology. What has not been given can scarcely be found in the pharmacopeia. My treatment of these cases has been very satisfactory, and I can unhesitatingly recommend it. Of course, the usual advice about excesses in eating, drinking and venery must be given. The avoidance of all excitement and emotional outbreaks, and a careful regard for the general health must be insisted upon.

The systematic use of the galvanic current is the most important element of treatment. The current should be weak, from ½ to 1½ milliampères, applied for a short time (one to three minutes) every other day. The cathode is applied at the angle of the lower jaw, first on one side and then on the other, while the anode is applied at the back of the neck. After ten to fifteen treatments a steadily progressing improvement is noted, which may last for years. This plan is recommended by such authorities as Erb, Benedict, Moritz Meyer and many others. The medicinal agents comprise, in first order, the slowly-increasing use of strophanthus, beginning with one drop of the tincture twice daily and increasing to ten. It is especially indicated where the tachycardia is well-pronounced. Ferguson, of Troy, has had uniformly good results with this drug, and has observed no relapses. Recognising tachycardia as an early symptom, he has been able to forestall the blossoming out of the disease through the use of strophanthus. Many of the ablest neurologists, as Gray, Gowers, Strümpell, Hirt and Seeligmüller, make no reference to it in describing the treatment of this disease, while Hammond, Oppenheim, Corning, Thompson and others rely upon strophanthus for controlling at least the tachycardia. As a tonic I know of nothing better, not only in this disease but in every disease of the nervous system, than the old-fashioned codliver oil. We have seen fortunes amassed by shrewd advertisers of proprietary medicines, the majority being tonics, have done our share to increase their profits, and yet at our very doors have in codliver oil a tonic, one tablespoonful of which is worth more than a pint of the ordinarily advertised nerve tonics. Drown your patients in the oil, nothing so restores a starving nerve-fiber or nerve-cell. For the insomnia, trional has done me yeoman service, and has never disappointed me, 5 to 10-grain powders producing a refreshing, healthful sleep.

Bromides for quieting temporarily the increased nerve activity, nux vomica and phosphoric acid for dyspeptic phenomena have given satisfaction.

The drinking of several pints of pure spring or distilled aerated water daily and a diet of nitrogenous foods mostly are important adjuncts of treatment.

Of late the administration of thyroid extract has attracted considerable attention, and many cases of improvement and recovery are on record. Personally I have not had any experience with this agent, but have seen cases and known of cases that have been benefited. Should I ever fail to attain results with the treatment laid down, I shall unhesitatingly try thyroid extract.

382 Virginia Street.

